# Equity, vulnerability and childhood immunization in Kenya and Uganda. A review of policy documents protocol.

**DOI:** 10.3310/nihropenres.13449.1

**Published:** 2023-11-24

**Authors:** Esther Awuor Owino, David Mafigiri, Dorcas Kamuya, Caroline Jones, Primus Chi

**Affiliations:** 1 Centre for Geographic Medicine Research (Coast), Kenya Medical Research Institute-Wellcome Trust Research Programme, Kilifi, Kenya; 2 Makerere University, Kampala, Central Region, Uganda; 3 Centre for Tropical Medicine and Global Health, Nuffield Department of Medicine, University of Oxford, Oxford, UK

**Keywords:** Equity, immunization, vulnerability, vaccine, vaccination, Kenya, Uganda, policy document review

## Abstract

**Introduction:**

Inequities in access and uptake of vaccines remains to be a challenge to the full realization of benefits they stand to offer. In the case of childhood immunization, improved full immunization coverage has been witnessed globally in the past decades but countries in sub-Saharan Africa have registered slow progress, with variations between/within countries. This has been attributed to several challenges/vulnerability factors. Consequently, current global efforts to promote access to immunization have focused on the issue of equity, which is now a strategic priority in key international policy documents such as the World Health Organization immunization agenda 2030 among others. It is therefore important to understand the context and efforts being made by individual countries to realize equity. We plan to undertake a systematic policy document review to understand the immunization context in Kenya and Uganda by examining the extent to which equity and vulnerability issues are framed and addressed in key health sector and policy documents in both countries.

**Methods:**

The systematic review will focus on key health sector and immunization policy documents in Uganda and Kenya between 2000 and 2023. Documents in the English language will be reviewed. Data sources will include official ministry of health websites for each country, websites of key international organizations working on immunization, general google searches and requesting for relevant documents from immunization stakeholders and officials in both countries. Data synthesis will follow a deductive and inductive approach. Findings will be presented in a descriptive format and in line with review objectives.

**Discussion:**

We will assess the extent to which equity and vulnerability issues are included and how they are defined in the health sector and immunization policy documents. In addition, the review will examine strategies proposed, planned and/or implemented to promote equitable access and uptake of immunization services in the two countries.

## Plain English Summary

Vaccines offer several benefits key among them being prevention of infectious disease outbreaks and deaths. Unfortunately, not everyone enjoys these benefits partly due to avoidable differences in reaching and using vaccines. In most African countries, there have been an increase in the number of those using vaccines over the years even though there are still differences between and within countries. In a bid to ensure that no one is left behind in reaching and using vaccines, most international organizations working around vaccines have now prioritized attaining fairness in use of vaccines and this commitment is reflected in international immunization policy documents. It is therefore important to examine how this is working out at country level. That is whether countries are also working towards ensuring that everyone is vaccinated and whether this is captured in their policy documents. This review therefore seeks to understand what is being done in Kenya and Uganda to ensure that everyone is reached with vaccines by examining key health sector and immunization policy documents.

The review will focus on key health sector and immunization policy documents published in the English language from 2000 to 2023. An online search will be conducted through relevant websites such as ministry of health websites for each country. In addition, documents that cannot be accessed *via* online sources will be requested from relevant stakeholders in the two countries. The documents will then be screened to determine those appropriate to the review objective. Data from selected documents will be extracted using a data extraction tool, analysed and presented in a descriptive format and in line with the objectives of the review.

## Introduction

Vaccines are some of the most effective public health interventions with a demonstrated capacity to prevent and/or control the outbreak and spread of infectious diseases ^1, 2^. Improved health outcomes globally can partly be attributed to the use of vaccines which have played a significant role in the reduction of morbidity and mortality arising from life threatening infectious diseases. According to the WHO, immunization against different infectious diseases contributes to the prevention of approximately four million deaths per year globally, hence increase in life expectancy. Moreover, disease prevention using vaccines can help to avert medical expenditures and other related costs such as loss of wages by caregivers during sickness episodes, travel time and transportation cost to seek treatment.

In the past decades, several concerted efforts have been put in place internationally to promote access and uptake of vaccines targeting different age groups. Key among these was the establishment of the Expanded Programme on Immunization (EPI) by WHO in 1974 and the Global Alliance for Vaccines and Immunization (GAVI) in 1999. These initiatives have so far led to improved childhood vaccine coverage across the globe. However, access and uptake between countries is still characterized by inequities especially in sub-Saharan African countries where majority of vaccine preventable diseases are experienced. Additionally, immunization coverage at national and sub-national levels in most African countries have stagnated and are disparate ^2– 6^. WHO estimates that more than 30 million of under five years children in Africa suffer from vaccine preventable diseases annually out of which 500,000 die from these diseases. This is almost equivalent to 58% of the global vaccine preventable diseases ^4^. Moreover, many of these children are those who haven’t received any dose of the recommended childhood vaccines (zero-dose children).

Current global efforts to promote immunization have focused on the issue of equity ^5, 7^. Equitable access to childhood vaccines is important since population groups or children who are unvaccinated or partially vaccinated are more vulnerable to outbreaks or re-emergence of infectious diseases ^5^. Equity in immunization has therefore been captured as a strategic priority in key international policy documents including the WHO immunization agenda 2021–2030 which aims at leaving no one behind, global vaccine action plan, Gavi’s 2021–2025 high-level strategy and the United Nations Children’s Fund (UNICEF) equity reference group for immunization ^5, 8^. It is therefore important to understand efforts being made by individual countries to reach vulnerable communities/ population groups and realize equity in immunization.

This policy analysis/document review therefore aims at understanding the immunization context in Kenya and Uganda by examining the extent to which equity and vulnerability issues are framed and addressed in health sector and immunization policy documents in the two countries. Specifically, the review will:

1. Examine the extent to which key health and immunization policy documents in Kenya and Uganda have included issues of vulnerability and (in)equity, how they are defined and proposed strategies to addressed them and promote uptake of vaccines.
2. Identify gaps and opportunities in the health sector and immunization policy documents.
3. Compare how issues of equity and vulnerability in the reviewed documents vary between Kenya and Uganda.

## Methods

### Eligibility criteria

The documents to be reviewed will include immunization policies, health sector strategies and guidelines, implementation and working documents such as annual and midterm reports, training manuals, government speeches among others. Only documents in English language will be included. Publication date for inclusion will be from 2000 (to correspond with the establishment of GAVI which has played a key role in promoting access to vaccines in most of the developing countries like Kenya and Uganda) to 2023. This will also help to provide a better understanding of the immunization context in both countries over the years as well as the efforts made to ensure that all children are vaccinated.

### Information sources

Documents to be reviewed will be retrieved from the following sources:

i. The official Kenyan and Ugandan Ministry of Health websites ( https://www.health.go.ke/
https://www.health.go.ug/)
ii. Websites of international and local organizations working around childhood immunization in Kenya and Uganda *e.g*., UNICEF, GAVI, WHO, Save the Children etc ( https://www.unicef.org/
https://www.gavi.org/;
https://www.who.int/;
https://kenya.savethechildren.net/)
iii. Requesting for relevant documents from the relevant stakeholders at MOH, Kilifi County department of health and donor and partner organizations.
iv. General google search

### Search strategy

The following key words will be used for general google searches as well as the specific websites listed above: Vaccin* and/or immuniz* AND/OR routine vaccin* and/or immuniz* and/or (In)equit* and/or vulnerab* AND/OR Expanded programme on immunization or EPI OR Kenya expanded programme on immunization or KEPI OR Uganda national expanded programme on immunization (UNEPI). In addition to the search terms listed above, documents will also be identified through the reference list of identified documents.

### Study records

***Data management and selection process.*** All documents retrieved from online sources will be exported and managed in Endnote v20 or Mendeley where duplicates will also be identified. The reviewer (EO) will manually screen the documents to identify those relevant to the review questions and meets the eligibility criteria. The screening will be based on the title of the document, a search of terms, including vulnerability, equity, immunization, and vaccination within the documents, and by reading through the executive summary of a document. The list of documents identified to be eligible for review will then be shared with the other reviewers (PC, CJ, DM and DK). The team will then meet to discuss about the appropriateness of the documents identified as eligible and a consensus reached on documents to be included for final review.

***Data collection process and data items.*** Data collection will be performed by EO using a data extraction template ^9^. Extracted data will then be shared with co-reviewers who will independently review it after which the team will meet to discuss and resolve any disagreement. The data extraction tool will be developed using an Excel spreadsheet. Each row of the tool will capture documents identified while the individual columns will capture the type of information to extract from the documents. Information to be extracted will include general information about the documents *e.g.,* the full title of the document, author(s), date of publication, purpose of the document, intended users/audience and a summary of issues addressed in the document. For documents about a specific policy, aspects of the policy analysis triangle will be captured. These will include context (why was the policy developed), content (what the policy is about), process (how the policy was brought forward and implemented), and actors (who are the persons involved in and influences the formulation and implementation of the policy). The second category of information to be extracted will include explicit and implicit information about equity, vulnerability, access, and uptake of immunization. To extract this type of data, full text review of documents that are entirely focused on immunization will be conducted while for documents that contain other information apart from immunization, only relevant sections will be read and data extracted in addition to the general information about the document.

### Review outcomes

The key outcomes for this review will be a description of how equity and vulnerability issues have been framed in immunization and health sector policy documents in Kenya and Uganda, strategies planned and/or implemented by each government with the aim of addressing vulnerabilities and promoting equity in access and uptake of vaccines, gaps and opportunities in the policy documents reviewed and how they compare between Kenya and Uganda.

### Risk of bias in individual studies

Not applicable

### Data synthesis

Data synthesis will follow a deductive and inductive approach. A modified version of UNICEF journey to health and immunization framework (JHIF) will be used to analyse the data deductively ^10– 12^. The JHIF outlines six key stages (before, during and after) in the journey to accessing and using immunization services. According to the framework, the first step entails knowledge, awareness and beliefs which in turn influences intent to get immunized, followed by preparation, cost and effort which leads to the point of service (immunization), experience of service and after service experience. The JHIF will therefore help to synthesize and understand which measures/strategies have been put in place to address the different stages in the journey to access and use childhood vaccines. The framework frameworks will also be useful in identifying gaps and opportunities in the existing strategies, policies and guidelines reviewed that can be exploited/tailored to improve access and uptake of childhood vaccines in Kenya and Uganda. In addition to the framework, an inductive approach will also be employed to capture new themes/ areas not captured in the framework. Findings from the review will be presented in a descriptive format in line with the review objectives.

### Meta-bias(es)

Not applicable

### Confidence in cumulative evidence

Not applicable

## Study status

Completion of this review is anticipated to be by September 2023. The study team is currently screening documents identified through online searches.

## Conclusion

The review intends to provide an understanding of the Kenyan and Ugandan context in regard to immunization policies. Specifically, it will focus on how equity and vulnerability issues are framed in policy documents in order to identify gaps and opportunities that can be harnessed to address vulnerabilities that influence access and uptake of vaccines. In addition, a comparison between Kenya and Uganda will be made and learnings drawn. We intend to explore the gaps identified through the review with relevant stakeholders in the two countries through primary research and contribute to improvement of the policy landscape around immunization through policy recommendations.

## Data Availability

Harvard Dataverse: Equity, vulnerability and childhood immunization in Kenya and Uganda: a systematic review of policy documents protocol. https://doi.org/10.7910/DVN/GZOQTL. This project contains the following extended data: Extended data file 1. PRISMA-P checklist (The checklist reports items addressed in this systematic review protocol). Extended data file 2. Data extraction template (This data extraction template will be used to manually extract relevant data from the documents included for analysis as per the review objectives) Data are available under the terms of the Creative Commons Attribution 4.0 International License (CC BY 4.0) A PRISMA-P checklist has been completed and uploaded on Harvard Dataverse. https://doi.org/10.7910/DVN/GZOQTL.
