# Vulnerability and childhood immunization in Kenya. A systematic review of policy documents protocol.

**DOI:** 10.3310/nihropenres.13449.3

**Published:** 2026-03-10

**Authors:** Esther Awuor Owino, David Mafigiri, Dorcas Kamuya, Caroline Jones, Primus Chi

**Affiliations:** 1 Centre for Geographic Medicine Research (Coast), Kenya Medical Research Institute-Wellcome Trust Research Programme, Kilifi, Kenya; 2 Makerere University, Kampala, Central Region, Uganda; 3 Centre for Tropical Medicine and Global Health, Nuffield Department of Medicine, University of Oxford, Oxford, UK

**Keywords:** Equity, immunization, vulnerability, vaccine, vaccination, Kenya, Uganda, policy document review

## Abstract

**Introduction:** Whereas improved full immunization coverage for childhood vaccines have been witnessed globally in the past decades, countries in sub-Saharan Africa have registered slow progress, with variations between and within countries. This has been attributed to several factors including socio-economic, individual and health system vulnerabilities. However, lacking is a documentation of how governments are conceptualizing and addressing these vulnerabilities. We therefore plan to systematically review policy documents to examine the extent to which vulnerability issues are conceptualized and addressed in the general health sector and immunization policy documents in Kenya.

**Methods:** The review will focus on the general health sector and immunization policy documents covering the period between 2000 and 2023. Documents in the English language will be reviewed. Data sources will include the official Ministry of Health website, websites of key international organizations working on immunization, checking reference lists of already identified documents, general Google searches and requesting relevant documents from stakeholders and officials. Data synthesis will follow an inductive approach, and findings will be presented in a descriptive format according to the review objectives.

**Discussion:** We will assess the extent to which vulnerability issues are included and how they are defined in the general health sector and immunization policy documents. In addition, the review will examine strategies proposed, planned and/or implemented to promote access and uptake of immunization services in Kenya. Using the review findings, we will formulate recommendations to share with health and Expanded Programme on Immunization (EPI) stakeholders at the national, county, and sub-county levels.

## Plain English Summary

Vaccines offer several benefits key among them being prevention of infectious disease outbreaks and deaths. Unfortunately, not everyone enjoys these benefits partly due to avoidable differences in reaching and using vaccines. In most African countries, there have been an increase in the number of those using vaccines over the years even though there are still differences between and within countries. In a bid to ensure that no one is left behind in reaching and using vaccines, most international organizations working around vaccines have now prioritized attaining fairness in use of vaccines and this commitment is reflected in international immunization policy documents. It is therefore important to examine how this is working out at country level. That is whether countries are also working towards ensuring that everyone is vaccinated and whether this is captured in their policy documents. This review therefore seeks to understand what is being done in Kenya and Uganda to ensure that everyone is reached with vaccines by examining key health sector and immunization policy documents.

The review will focus on key health sector and immunization policy documents published in the English language from 2000 to 2023. An online search will be conducted through relevant websites such as ministry of health websites for each country. In addition, documents that cannot be accessed *via* online sources will be requested from relevant stakeholders in the two countries. The documents will then be screened to determine those appropriate to the review objective. Data from selected documents will be extracted using a data extraction tool, analysed and presented in a descriptive format and in line with the objectives of the review.

## Introduction

Improved health outcomes globally can partly be attributed to the use of vaccines, which have played a significant role in reducing morbidity and mortality arising from life-threatening infectious diseases. ^1– 3^ Over the past decades, several concerted international efforts have been put in place to promote access to and uptake of vaccines targeting different age groups. Key among these was the establishment of the Expanded Programme on Immunization (EPI) by WHO in 1974 and the Global Alliance for Vaccines and Immunization (GAVI) in 1999. These initiatives have so far led to improved childhood vaccination coverage worldwide. However, access and uptake between countries is still characterized by inequities, especially in sub-Saharan Africa where the majority of vaccine-preventable diseases are experienced. ^4– 6^ WHO estimates that more than 30 million children under five years old in Africa suffer from vaccine-preventable diseases annually, out of which 500,000 die from these diseases. This is almost equivalent to 58% of the global burden of vaccine-preventable diseases. ^2^ Moreover, many of these children haven’t received any dose of the recommended childhood vaccines. Additionally, immunization coverage at national and sub-national levels in most African countries has stagnated and is disparate. ^6^ For instance, in Kenya, the 2022 Kenya Demographic and Health Survey (KHDS) reported full vaccination coverage with basic vaccines at 80%, with Vihiga County reporting the highest coverage of 96% while Garissa County had the lowest coverage of 23%. Furthermore, only 55% of children aged between 12 and 23 months and 38% of those in the age bracket of 24–35 months had been fully vaccinated as per the national immunization schedule. ^7^

These disparities not only reflect vulnerabilities influencing access and uptake of vaccines, but also make children experiencing the disparities vulnerable to vaccine-preventable diseases, thereby forming hotspots for future disease outbreaks. Vulnerability has been defined in different ways, as it is rooted in several disciplines. As a concept, vulnerability has been recognised as an inherent human condition due to our embodiment as human beings and affective nature. In addition, vulnerability has been conceptualized as concrete situations arising from multiple, overlapping, dynamic, contextual and multidimensional factors including individual, social and structural dimensions. For instance, Grabovschi et al. (2013) in their review of mapping the concept of vulnerability related to healthcare disparities operationalized vulnerability as an increased susceptibility to health and health care disparities due to several factors at the individual and environmental level. ^8^ They defined individual-level factors as either inborn (sex, race, genetic predispositions to disease) or acquired (trauma, diseases, lifestyle). On the other hand, environmental factors included those in the immediate physical environment or the broader socio-economic environment.

Several reviews of published literature have been conducted to highlight vulnerabilities that influence the disparities in access and uptake of childhood vaccines. ^9, 10^ For instance, in Kenya, previous studies have shown that children’s vulnerability to vaccine-related disparities is influenced by geographical, household, and mothers’ characteristics. ^11, 12^ However, reviews on how these vulnerability issues are addressed in grey literature, such as government policies, are lacking. Yet this understanding is not only important but also necessary for the identification of gaps and the formulation of appropriate strategies to ensure access and uptake. We therefore intend to systematically review general health sector and immunisation-specific policy documents in Kenya to examine the extent to which vulnerability issues are framed [ 2] and addressed generally and in the context of immunization. Specifically, the review will: (1) describe how vulnerability is defined and components considered in the general health sector and immunisation-specific policy documents, and (2) examine strategies proposed in the policy documents to address vulnerability issues in general and in the context of immunization.

## Methods

### Inclusion criteria

The documents to be reviewed will include general health sector and immunization specific policies, guidelines and strategic plans. Only documents in the English language will be included given that English is the primary language for official government policy documentation, especially for national and county-level strategies, policies and plans in Kenya. In addition, including only English policy documents is necessary to ensure consistent interpretation of the policy documents. Publication date for inclusion will be from 2000 (to correspond with the establishment of GAVI, which has played a key role in promoting access to vaccines in most of the developing countries like Kenya) to 2023.

### Information sources

Documents to be reviewed will be retrieved from the following sources:

i. The official Kenyan Ministry of Health website ( https://www.health.go.ke/).
ii. Websites of international and local organizations working around childhood immunization in Kenya *e.g.*, UNICEF, GAVI, WHO, Save the Children etc ( https://www.unicef.org/; https://www.gavi.org/; https://www.who.int/; https://kenya.savethechildren.net/)
iii. Requesting for relevant documents from the relevant stakeholders at MOH, Kilifi County department of health and donor and partner organizations.
iv. Reference list of included documents.
v. General google search.

Google search results will be screened for relevance based on alignment with the study objectives and inclusion criteria. Only documents published by official government institutions and recognized international health organizations will be prioritized.

### Search strategy

The following keywords will be used for general Google searches as well as the specific websites listed above: Vaccin* and/or immuniz* AND/OR routine vaccin* and/or immuniz* and/or vulnerab* AND/OR Expanded programme on immunization or EPI OR Kenya expanded programme on immunization or KEPI. Sample search strings will include: (Vaccin* OR immuniz*) AND Kenya; (routine vaccin* OR routine immuniz*) AND Kenya; (Vaccin* OR immuniz*) AND (vulnerab* OR marginalized) AND Kenya; (routine vaccin* OR routine immuniz*) AND (“Expanded Programme on Immunization” OR EPI OR KEPI).

In addition to the search strategy presented above, documents will also be identified from the reference lists of the identified documents.

### Study records

***Data management and selection process.*** All documents retrieved from online sources will be exported and managed in Endnote v20 or Mendeley where duplicates will also be identified. The reviewer (EO) will manually screen the documents to identify those relevant to the review questions and that meet the eligibility criteria. The screening will be based on the title of the document, a search of terms, including vulnerability, immunization, and vaccination within the documents, and by reading through the executive summary of a document. We don’t intend to apply formal inter-rater reliability measures (e.g., Cohen’s Kappa); however, to ensure consistency and quality control, screening decisions and the list of documents identified as eligible for review will be shared with the PC, CJ, DM and DK for verification. EO and PC, CJ, DM and DK will then meet to discuss the appropriateness of the documents identified as eligible. During these meetings, any disagreements will be discussed, and a consensus will be reached on documents to be included for final review. The process of selecting relevant documents for review will be summarized using a PRISMA-style flow diagram, detailing the number of documents identified, screened, included, and excluded, along with reasons for exclusion.

***Data collection process and data items.*** Data collection will be performed by EO using a data extraction template. ^13^ The extracted data will then be shared with co-reviewers, who will independently review it. Afterwards, the team will meet to discuss and resolve any disagreements. The data extraction tool will be developed using an Excel spreadsheet. Each row of the tool will capture the identified documents, while the individual columns will capture the types of information to extract from them. Information to be extracted will include general information about the documents, *e.g.,* the full title of the document, author(s), date of publication, purpose of the document, intended users/audience and a summary of issues addressed in the document. The second category of information to be extracted will include how vulnerability is defined, who is considered vulnerable and strategies to address the needs of those considered vulnerable. To extract this type of data, a full-text review of documents that are entirely focused on immunization will be conducted, while for documents that contain other information apart from immunization, only relevant sections will be read and data extracted, in addition to the general information about the document.

### Review outcomes

The key outcomes for this review will be a description of how vulnerability issues have been conceptualized in immunization and health sector policy documents in Kenya, strategies planned and/or implemented by the Kenyan government to address health-related vulnerabilities and those specific to access and uptake of vaccines in the policy documents reviewed. Findings from this review will be shared with relevant stakeholders through policy briefs and dissemination meetings and explored further through primary research focusing on vulnerabilities influencing childhood vaccination in Kenya.

### Risk of bias in individual studies

Although every effort will be made to secure all relevant documents for the review, we recognize that we may miss unpublished or documents that are still being drafted. Also the review findings will be limited to data drawn from **policy** documents which fall within the inclusion criteria (only English documents, and those published from 2000–2023).

### Data synthesis

Data synthesis will follow an inductive approach where the extracted data on vulnerability will be read line by line and appropriate codes applied in Nvivo 12 software. Data will then be organized thematically to address the review objectives: how vulnerability issues are conceptualized/defined, aspects considered in the documents and strategies proposed to address vulnerability issues. The themes developed will be reviewed and revised through comparison across documents, and any discrepancies will be discussed amongst reviewers until a consensus is reached.

### Meta-bias(es)

Not applicable.

### Confidence in cumulative evidence

Not applicable.

## Study status

Completion of this review is anticipated to be by September 2023. The study team is currently screening documents identified through online searches.

## Conclusion

The review intends to provide an understanding of vulnerability issues in the context of health and immunization in Kenya. Specifically, it will focus on how vulnerability issues are framed in policy documents to identify gaps and opportunities that can be harnessed to address vulnerabilities that influence access and uptake of vaccines. We intend to explore the gaps identified through the review with relevant stakeholders through primary research and contribute to the improvement of the policy landscape around immunization by sharing the findings through policy briefs and dissemination meetings with health and EPI stakeholders at national, county and sub-county levels.

## Data Availability

Harvard Dataverse: Equity, vulnerability and childhood immunization in Kenya and Uganda: a systematic review of policy documents protocol. https://doi.org/10.7910/DVN/GZOQTL. This project contains the following extended data:
• Extended data file 1. PRISMA-P checklist (The checklist reports items addressed in this systematic review protocol). • Extended data file 2. Data extraction template (This data extraction template will be used to manually extract relevant data from the documents included for analysis as per the review objectives) Data are available under the terms of the Creative Commons Attribution 4.0 International License (CC BY 4.0). A PRISMA-P checklist has been completed and uploaded on Harvard Dataverse. https://doi.org/10.7910/DVN/GZOQTL.

## Footnotes

^2^
Here framing refers to how vulnerability is defined and operationalized as well as components considered.
