# Supplementary material for: Vulnerability and childhood immunization in Kenya. A systematic review of policy documents protocol
Source: NIHR Open Res. 2026 Mar 10;3:64. Originally published 2023 Nov 24. [Version 3] doi: 10.3310/nihropenres.13449.3 (PMC12586977; doi:10.3310/nihropenres.13449.3)
Supplement: Peer review history

# Reviewer report: Reviewer response for version 3

**DOI:** 10.3310/nihropenres.15530.r39926

**Published:** 2026-04-06

**Authors:** Morufu Olalekan Raimi

**Affiliations:** 1 Federal University Otuoke, Otuoke, Nigeria

**Manuscript Number:** NIHR Open Research 2026, 3:64 (Version 3)

**Title:** Vulnerability and Childhood Immunization in Kenya: A Systematic Review of Policy Documents Protocol

**Recommendation:** Minor Revision

**Reviewer:** Dr. Morufu Olalekan Raimi, PhD (Academic Editor, PLOS One – Environmental Epidemiology)

**Review Date:** 2026-04-04

**Reviewer Comments**

 Dear Authors,

 Thank you for submitting this revised protocol for a systematic review of policy documents examining how vulnerability is conceptualized and addressed in Kenya’s health sector and immunization policies. 

 The protocol is clearly written, methodologically sound, and addresses an important gap in the literature. The focus on policy documents, rather than published peer-reviewed studies, is a distinctive and valuable contribution, as national policies shape resource allocation, program design, and implementation strategies. The revisions made in response to previous reviewer comments have substantially improved the protocol’s clarity and rigor. The study is timely given persistent sub-national disparities in immunization coverage in Kenya (e.g., 96% in Vihiga vs. 23% in Garissa). 

 The authors’ decision to narrow the scope from two countries (Kenya and Uganda) to Kenya alone is appropriate and enhances feasibility. The addition of a footnote clarifying that this is a review of policy documents (not clinical trials) is helpful, as is the inclusion of specific search strings, a data extraction template, and acknowledgment of potential biases. However, several issues remain that require attention before the protocol can be accepted for publication. These relate to conceptual clarity around “vulnerability,” the absence of a quality appraisal framework for policy documents, insufficient detail on how implicit mentions of vulnerability will be identified, and the lack of a clear dissemination and stakeholder engagement plan. Below I provide a detailed, section-by-section critique.

**I. Title**

 The revised title is clear, specific, and accurately reflects the scope. No revision needed.

**II. Abstract**

 The abstract provides a good overview but lacks several key elements expected in a protocol.

**Issues:**

- The methods state that “documents in the English language will be reviewed” but do not specify the number of documents expected or the search date range (2000-2023 is mentioned in the main text but not the abstract). Add the date range.
- The abstract does not mention how data will be synthesized (inductive thematic analysis using NVivo). Add a brief sentence.
- The discussion states that “we will formulate recommendations” but does not specify the intended audience or dissemination format (policy briefs, stakeholder meetings). Add this.
- The plain English summary remains overly long and repetitive. For example, the second paragraph restates information from the first. Condense.

**Recommendation:** Add date range, synthesis method, and dissemination plans to the abstract. Shorten the plain English summary by removing redundant sentences.

**III. Introduction**

 The introduction has been improved with the addition of recent coverage data (KDHS 2022) and a definition of vulnerability. However, several issues persist.

**Issues:**

1. **Definition of vulnerability (lines 4-5, page 4):** The authors provide a definition citing Grabovschi et al. (2013). However, this definition is presented as a single block of text without distinguishing between the authors’ own operationalization and the cited source. Clarify which parts are directly from Grabovschi and which are the authors’ synthesis.
2. **Framing footnote (footnote 2, page 4):** The footnote states that “framing refers to how vulnerability is defined and operationalized as well as components considered.” This is helpful, but the term “operationalized” is not explained in the context of policy document analysis. What does operationalization look like in a policy document? (e.g., specific budget lines, targeted programs, monitoring indicators). Expand slightly.
3. **Gap statement (lines 6–7, page 4):** The authors state that “reviews on how these vulnerability issues are addressed in grey literature, such as government policies, are lacking.” This is accurate, but the authors should acknowledge that previous reviews of published literature (e.g., Bangura et al., 2020; Galadima et al., 2021) have identified vulnerability factors, and the present review builds on this by examining policy responses. A brief bridging sentence would strengthen the rationale.
4. **Country focus:** The title and abstract now focus exclusively on Kenya, but the introduction still mentions “Kenya and Uganda” in one instance (line 8, page 4: “We therefore intend to systematically review general health sector and immunisation-specific policy documents in Kenya”). The Uganda reference has been removed from the title and methods, but the plain English summary (page 2) still states “This review therefore seeks to understand what is being done in Kenya and Uganda.” This inconsistency must be corrected. The plain English summary should refer only to Kenya.

**Recommendation:** Clarify the vulnerability definition attribution, expand the framing footnote, add a bridging sentence linking to previous reviews of published literature, and correct the plain English summary to reflect the single-country focus.

**IV. Methods**

 The methods section is largely well-specified, but several areas require additional detail or clarification.

**4.1 Inclusion criteria (page 5)**

**Issues:**

- The rationale for excluding Kiswahili-language documents is provided (“English is the primary language for official government policy documentation”). This is reasonable, but the authors should acknowledge that some county-level documents or community-facing materials may be in Kiswahili or local languages, and their exclusion is a limitation. Add this to the limitations section (currently in the conclusion, but expand).
- The date range (2000-2023) is justified by the establishment of GAVI. However, the review is being conducted in 2026 (manuscript received March 2026). The authors should clarify whether documents published in 2024-2025 will be included, or if the search is closed as of 2023. If the latter, state this explicitly.

**Recommendation:** Add a limitation acknowledging exclusion of non-English documents, and clarify whether the search period remains 2000-2023 or has been updated.

**4.2 Information sources and search strategy (page 5)**

**Issues:**

- The authors state that “Google search results will be screened for relevance based on alignment with the study objectives and inclusion criteria. Only documents published by official government institutions and recognized international health organizations will be prioritized.” This is appropriate, but the protocol does not specify how many pages of Google results will be reviewed (e.g., first 10 pages, or until saturation). Specify.
- The sample search strings are provided, which is excellent. However, the strings use truncation (e.g., “Vaccin*”) but do not specify whether the search is case-sensitive or whether phrase searching (quotation marks) will be used. Add brief technical specifications.

**Recommendation:** Specify Google search depth (e.g., “first 10 pages or until no new relevant documents appear”) and add basic search syntax details.

**4.3 Study records and selection process (pages 5-6)**

**Issues:**

- The authors state that “we don’t intend to apply formal inter-rater reliability measures (e.g., Cohen’s Kappa); however, to ensure consistency and quality control, screening decisions and the list of documents identified as eligible for review will be shared with the PC, CJ, DM and DK for verification.” This is acceptable for a policy document review, but the protocol should specify what proportion of documents will be independently screened by a second reviewer (e.g., “all documents identified as eligible will be reviewed by a second team member”). Current wording suggests only that screening decisions are “shared” after the fact, not that independent double-screening occurs. Clarify.

**Recommendation:** Specify whether a second reviewer will independently screen a subset (e.g., 20%) or all eligible documents, and how disagreements will be documented.

**4.4 Data collection and data items (pages 5-6)**

**Issues:**

- The data extraction template is included as extended data, which is commendable. However, the description of what will be extracted for vulnerability (“how vulnerability is defined, who is considered vulnerable and strategies to address the needs of those considered vulnerable”) does not address *implicit* mentions of vulnerability. A policy document may not use the word “vulnerable” but may describe “hard-to-reach populations,” “nomadic groups,” “urban poor,” or “zero-dose children.” The authors should specify how such implicit references will be identified and coded. This was raised by Reviewer Raimi and only partially addressed in the response (“we have spelt out the specific information”). The current text does not yet include explicit guidance on implicit mentions. Add a sentence: “Implicit references to vulnerability (e.g., descriptions of geographic isolation, poverty, maternal illiteracy, or marginalized ethnic groups without using the term ‘vulnerable’) will also be extracted and coded.”

**Recommendation:** Add explicit guidance on identifying and extracting implicit vulnerability mentions.

**4.5 Risk of bias / limitations (page 6)**

**Issues:**

- The authors have added a statement acknowledging that “we may miss unpublished or documents that are still being drafted” and that findings are limited to documents meeting inclusion criteria. This is good. However, the protocol does not address the *quality* of included policy documents. Unlike peer-reviewed articles, policy documents vary in quality (e.g., some may be drafts, others may lack implementation details). The authors state in their response to Reviewer Spencer that “we only aimed to examine the content of the documents in regard to vulnerability since the documents were not vulnerability-specific.” This is acceptable, but the protocol should still specify that no formal quality appraisal will be applied and justify this decision. Add a brief sentence.

**Recommendation:** Add a sentence stating that formal quality appraisal of policy documents will not be conducted, as the review aims to describe content rather than assess evidence quality, and note this as a limitation.

**4.6 Data synthesis (page 6)**

**Issues:**

- The authors state that “data synthesis will follow an inductive approach where the extracted data on vulnerability will be read line by line and appropriate codes applied in Nvivo 12 software.” This is clear. However, the protocol does not specify how themes will be developed (e.g., will coding be deductive based on the research questions, or purely inductive?). A hybrid approach is common. Specify.
- The authors state that “themes developed will be reviewed and revised through comparison across documents, and any discrepancies will be discussed amongst reviewers until a consensus is reached.” This is appropriate, but the protocol should specify how many reviewers will be involved in coding (one? two?).

**Recommendation:** Specify whether coding is inductive, deductive, or hybrid, and state the number of reviewers involved in coding and theme development.

**V. Conclusion (page 6)**

 The conclusion is brief and appropriately states the intended use of findings.

**Issues:**

- The conclusion states that “we intend to explore the gaps identified through the review with relevant stakeholders through primary research.” This is vague. What type of primary research? Interviews? Workshops? Policy dialogues? Specify.
- The conclusion does not mention the timeline for completion (the “Study status” section on page 6 states “Completion of this review is anticipated to be by September 2023.” That date has passed. Update the timeline or remove the statement.

**Recommendation:** Specify the planned primary research method (e.g., “key informant interviews” or “stakeholder workshops”) and update or remove the obsolete timeline.

**VI. Extended Data and Reporting (page 6)**

 The extended data (PRISMA-P checklist and data extraction template) are appropriately referenced. However:

**Issue:**

- The PRISMA-P checklist is included, but the protocol does not state whether a PRISMA flow diagram will be included in the final review (not the protocol). This is fine, but the authors should confirm that the final review will include a flow diagram.

**Recommendation:** Add a statement that the final review will include a PRISMA flow diagram documenting document identification, screening, inclusion, and exclusion.

**VII. References**

 The references are appropriate and up-to-date. However:

**Issue:**

- Reference 7 (KNBS, 2023) - the citation is incomplete. The full title of the report is “Kenya Demographic and Health Survey 2022.” Add the publisher and URL if available.
- Reference 13 (Owino *et al.,* 2023) - this is the earlier version of this protocol. This is fine, but the authors should ensure that all in-text citations to this reference are updated to the current version.

**Recommendation:** Complete reference 7 and verify in-text citations.

**VIII. Language and Presentation**

 The manuscript is well-written, but several minor issues remain.

**Specific errors:**

- Page 1, line 1: “Centre for Geographic Medicine Research (Coast)” - the parentheses around “Coast” are fine, but ensure consistency with the institutional name as used elsewhere.
- Page 2, plain English summary, line 4: “avoidable differences in reaching and using vaccines” - consider “avoidable differences in access to and uptake of vaccines” for clarity.
- Page 2, plain English summary, line 8: “attaining fairness in use of vaccines” - consider “attaining equity in vaccine access and uptake.”
- Page 4, footnote 2: The footnote is placed after the word “framed.” The explanation is clear, but the footnote marker should be superscript. Format accordingly.
- Page 5, line 5: “publication date for inclusion will be from 2000… to 2023” - add “the period” before “2000.”
- Page 5, search strategy: The sample search strings are presented without quotation marks or formatting. Consider presenting them in a bulleted list for clarity.
- Page 6, line 4: “we don’t intend” - use “do not” for formal academic writing.
- Page 6, line 6: “PC, CJ, DM and DK” – these initials are not defined in the text (they appear to be the co-authors). Define them as “PC (Primus Chi), CJ (Caroline Jones), DM (David Mafigiri), and DK (Dorcas Kamuya).”

**Recommendation:** Minor copy-editing for grammar, consistency, and formal style.

**IX. Overall Assessment and Recommendation**

 This protocol addresses an important and under-researched question: how national health and immunization policies in Kenya conceptualize and respond to vulnerability. The focus on policy documents rather than published literature is a distinctive contribution, as policies directly shape implementation. The protocol is well-structured, the methods are largely appropriate, and the authors have responded thoughtfully to previous reviewer comments. However, several issues require attention before the protocol can be accepted. The most critical are: (1) inconsistency between the title/abstract (Kenya only) and the plain English summary (Kenya and Uganda), which must be corrected; (2) insufficient guidance on identifying implicit mentions of vulnerability in policy documents; (3) lack of clarity on whether formal quality appraisal will be applied to policy documents; (4) the need to specify the number of reviewers involved in screening and coding; and (5) the need to update or remove the obsolete timeline (September 2023). These revisions are minor in scope and can be completed without changing the overall design or analytic approach.

**Recommendation:** Minor Revision.

**X. Specific Action Items for Authors**

1. **Introduction / Plain English summary:** Correct the inconsistency regarding Uganda. The plain English summary should refer only to Kenya.
2. **Methods - Inclusion criteria:** Acknowledge exclusion of non-English documents as a limitation; clarify whether the search period remains 2000-2023 or has been extended.
3. **Methods - Search strategy:** Specify Google search depth (e.g., “first 10 pages”); add basic search syntax details.
4. **Methods - Study selection:** Specify whether a second reviewer will independently screen a subset of documents and how disagreements will be resolved.
5. **Methods - Data extraction:** Add explicit guidance on identifying implicit mentions of vulnerability (e.g., “hard-to-reach populations,” “nomadic groups,” “zero-dose children”).
6. **Methods - Data synthesis:** Specify whether coding is inductive, deductive, or hybrid; state the number of reviewers involved in coding.
7. **Methods - Risk of bias / quality appraisal:** Add a sentence stating that formal quality appraisal of policy documents will not be conducted, with justification.
8. **Conclusion:** Specify the planned primary research method (e.g., interviews, workshops) and update or remove the obsolete timeline.
9. **References:** Complete reference 7 (KNBS, 2023).
10. **Language:** Minor copy-editing for consistency and formal style; define co-author initials.

I look forward to seeing the revised protocol. This work has the potential to inform policy and practice aimed at reducing immunization inequities in Kenya.

 Sincerely,

 Dr. Morufu Olalekan Raimi, PhD

 Academic Editor (Environmental Epidemiology)

 PLOS One

Is the study design appropriate for the research question?

Yes

Is the rationale for, and objectives of, the study clearly described?

Yes

Are sufficient details of the methods provided to allow replication by others?

Yes

Are the datasets clearly presented in a useable and accessible format?

Yes

Reviewer Expertise:

Environmental Epidemiology; Climate Change and Health; Pollution Control Management; Public Health; Groundwater Quality

I confirm that I have read this submission and believe that I have an appropriate level of expertise to confirm that it is of an acceptable scientific standard.

# Reviewer report: Reviewer response for version 3

**DOI:** 10.3310/nihropenres.15530.r39927

**Published:** 2026-03-12

**Authors:** Nicholas Spencer

**Affiliations:** 1 University of Warwick, Coventry, England, UK

The authors have revised the paper in line with my suggestions and those of other reviewers. I am satisfied that the revisions have improved the protocol.

Is the study design appropriate for the research question?

Partly

Is the rationale for, and objectives of, the study clearly described?

Yes

Are sufficient details of the methods provided to allow replication by others?

Partly

Are the datasets clearly presented in a useable and accessible format?

Not applicable

Reviewer Expertise:

Equity research

I confirm that I have read this submission and believe that I have an appropriate level of expertise to confirm that it is of an acceptable scientific standard.

# Reviewer report: Reviewer response for version 2

**DOI:** 10.3310/nihropenres.15217.r36147

**Published:** 2025-11-04

**Authors:** Morufu Olalekan Raimi

**Affiliations:** 1 Federal University Otuoke, Otuoke, Nigeria

**A. GENERAL EVALUATION**

 This protocol addresses an urgent global health equity issue, the inclusion (or exclusion) of vulnerability in policy frameworks guiding childhood immunization in Kenya. It is conceptually timely and methodologically appropriate for shaping more equitable vaccine strategies. The revised version improves upon the original scope and aligns well with the associated primary study. However, several sections still suffer from:

- Insufficient methodological detail for full replication,
- A lack of conceptual clarity around “vulnerability,”
- Overuse of generic phrasing without sufficient policy analysis theory grounding,
- Structural inconsistencies (e.g., ambiguous timelines, redundancy in explanation).

This review presents detailed paragraph-level feedback, followed by overall recommendations.

**B. DETAILED SECTION-BY-SECTION REVIEW**

**1. Title and Abstract**

- The revised title is clearer and better aligned with the focus on Kenya and “vulnerability.” Abstract introduces rationale, methods, and aims adequately.

- The term “systematic review” remains potentially misleading for policy documents. Though addressed in previous peer reviews, a footnote or clarification on the nature of this “review” (policy document analysis vs. intervention or clinical trial) may still be helpful.
- The sentence “...this has been attributed to several challenges/vulnerability factors” is vague. Use more precise terms (e.g., geographic inequality, socio-economic status, maternal education, etc.). Author should consider “systematic policy review” or “document analysis” as part of the subtitle.

- Add 1-2 key expected outputs (e.g., frameworks, recommendations) in the abstract.

**2. Plain English Summary**

- Simplifies technical content for lay readers effectively and gives a clear sense of relevance and practical application. Contains overly long and repetitive sentences.

- The last paragraph restates the same information from the abstract almost verbatim. **Example:** “...examine how this is working out at country level...whether this is captured in their policy documents.” This can be shortened and rephrased for clarity. Author should trim redundancy; group benefits, equity aims, and method into clearer subsections as well as add clarity on the population of interest (e.g., age ranges, vulnerable groups).

**3. Introduction**

- Strong justification for immunization as a public health strategy and use of global data and WHO sources improves credibility. Many sentences are generic and overstate well-known facts (e.g., “Vaccines...prevent disease outbreaks and deaths”). Author should cite specific immunization coverage stats from Kenya to better localize the narrative (e.g., coverage in counties like Turkana vs. Nairobi).

- Cites previous reviews and identifies literature gaps effectively. The claim: “how these vulnerability issues are framed...in grey literature...is lacking” is weak without citing national health policies or strategies reviewed in prior studies. Author should cite at least 1-2 key documents (e.g., Kenya Health Policy 2014–2030) to support this gap. Expand on what “framing” means, define whether it's inclusion, prioritization, or operationalization of vulnerability.

**4. Methods**

**Inclusion Criteria:**

- Temporal scope (2000–2023) is justified and aligns with GAVI-era reforms and language and type of documents are clearly listed. The rationale for focusing solely on English-language documents could be expanded upon given potential inclusion of Kiswahili policy texts.

**Information Sources & Search Strategy:**

- Multiple source types are considered, from MOH websites to gray literature and personal contacts. No detail is provided on how the Google searches will be validated for relevance, or how source credibility will be assessed. Author should add a sample search string (already suggested by a previous reviewer) and include a visual flow diagram (e.g., PRISMA-style chart) in the final paper.

 

**Study Records, Data Management & Selection:**

- Use of EndNote/Mendeley and consensus screening is appropriate. No mention of whether inter-rater reliability or agreement measures (e.g., Cohen’s Kappa) will be used. Author should clarify the criteria for resolving disagreements during screening as well as specify who will perform quality control and ensure consistency.

 

**Data Collection and Extraction:**

- Inclusion of a data extraction template is commendable and enhances transparency.

- The explanation of what constitutes “explicit and implicit” vulnerability-related information is vague. Author should define indicators of implicit vulnerability (e.g., mention of remote populations, nomadic groups, maternal illiteracy) and consider aligning categories with a policy analysis framework such as Walt & Gilson’s policy triangle.

**Review Outcomes:**

  

- Clearly articulated: framing, strategies, gaps, and opportunities. “Gaps and opportunities” are vague without examples of potential policy levers (e.g., funding, service delivery). Author should mention potential uses of findings (e.g., stakeholder engagement, policy briefs).

 

**5. Data Synthesis**

- Inductive thematic analysis is appropriate for qualitative document review. No mention of NVivo or qualitative coding software to manage complex themes. Author should consider using software like NVivo or Atlas.ti for theme development and outline how themes will be validated or revised.

 

**6. Ethics & Bias Consideration**

- Noted as “Not applicable” for risk of bias/meta-bias. While true for empirical bias, the risk of selection bias in policy document inclusion exists and is not addressed. Author should add a paragraph in the discussion acknowledging this limitation e.g., exclusion of unpublished or draft policies.

 

**7. Conclusion**

- Emphasizes intended use of findings to influence policy. Too generic. Does not outline how findings will be shared, with whom, and for what purpose. **Hence, **Author should add specifics: “We will share findings with the Kenya EPI taskforce, civil society actors, and Ministry of Health policymakers through workshops and briefings.”

 

**8. Extended Data & Reporting**

- Inclusion of PRISMA-P and a data extraction template shows protocol rigor. Author should ensure that the final publication also includes:

- PRISMA flow diagram.
- A sample of coded policy excerpts in supplementary material.

 

**C. OVERALL STRENGTHS**

- Responds well to previous reviews and narrows its scope effectively.
- Clearly structured protocol with transparent data collection and extraction plans.
- Solid foundation for future primary research on immunization access in Kenya.

 

**D. AREAS NEEDING REVISION**

**Area**

**Recommendations**

 Conceptual Clarity

 Define “vulnerability” more rigorously; use a guiding policy framework (e.g., WHO’s equity lens).

 Search Strategy

 Include a sample string and Google search filtering criteria.

 Data Extraction & Coding

 Add criteria for identifying implicit mentions; consider coding software.

 Risk of Bias

 Discuss selection bias from document inclusion and relevance thresholds.

 Conclusion

 Specify dissemination pathways and intended policy impact more explicitly.

 Recommendation: Moderate Revision

Is the study design appropriate for the research question?

Yes

Is the rationale for, and objectives of, the study clearly described?

Yes

Are sufficient details of the methods provided to allow replication by others?

Yes

Are the datasets clearly presented in a useable and accessible format?

Yes

Reviewer Expertise:

Environmental Epidemiology; Climate Change and Health; Pollution Control Management; Public Health; Groundwater Quality

I confirm that I have read this submission and believe that I have an appropriate level of expertise to confirm that it is of an acceptable scientific standard, however I have significant reservations, as outlined above.

# Response

**Published:** 2026-02-20

**Authors:** Esther Owino

**Affiliations:** KEMRI Wellcome Trust Research Programme, Kenya

Thank you very much Dr Morufu Olalekan Raimi, for taking the time to review our manuscript. We truly appreciate your valuable and useful feedback and comments. ****Below, please find our responses to the issues you raised. 

**Title and abstract**

 1. The revised title is clearer and better aligned with the focus on Kenya and “vulnerability.” Abstract introduces rationale, methods, and aims adequately. The term “systematic review” remains potentially misleading for policy documents. Though addressed in previous peer reviews, a footnote or clarification on the nature of this “review” (policy document analysis vs. intervention or clinical trial) may still be helpful.

 Response: We have added a footnote to clarify that this is a review of policy documents and not published peer-reviewed literature.

 2. The sentence “...this has been attributed to several challenges/vulnerability factors” is vague. Use more precise terms (e.g., geographic inequality, socio-economic status, maternal education, etc.). Author should consider “systematic policy review” or “document analysis” as part of the subtitle.

 Response: We have included more precise terms (socio-economic, individual and health system vulnerabilities)

 3. Add 1-2 key expected outputs (e.g., frameworks, recommendations) in the abstract.

 Response: We have indicated that using the review findings, we will formulate recommendations to be shared with health and EPI stakeholders at the national, county and sub-county levels.

**Plain English Summary**

 4: Simplifies technical content for lay readers effectively and gives a clear sense of relevance and practical application. Contains overly long and repetitive sentences.

 Response: We have revised the plain English summary to be more precise

**Introduction**

 5. Many sentences are generic and overstate well-known facts (e.g., “Vaccines...prevent disease outbreaks and deaths”). Author should cite specific immunization coverage stats from Kenya to better localize the narrative (e.g., coverage in counties like Turkana vs. Nairobi).

 Response: To illustrate the disparities within the country, we have provided the recent vaccine coverage data from the 2022 Kenya Demographic and Health Survey (KHDS).

 6. Expand on what “framing” means, define whether it's inclusion, prioritization, or operationalization of vulnerability.

 Response: A footnote has been added to expand on what we mean by “framing”. In this protocol, framing refers to how vulnerability is defined and operationalized as well as components considered.

**Methods**

 7. The rationale for focusing solely on English-language documents could be expanded upon given potential inclusion of Kiswahili policy texts.

 Response: We have expanded on the rationale of focusing on the English documents, which is because English is the primary language for official government policy documentation, especially for national and county-level strategies, policies and plans in Kenya. In addition, we have included only English policy documents to ensure consistent interpretation of the policy documents.

 8. No detail is provided on how the Google searches will be validated for relevance, or how source credibility will be assessed. Author should add a sample search string (already suggested by a previous reviewer) and include a visual flow diagram (e.g., PRISMA-style chart) in the final paper.

 Response: To validate Google searches, Google search results will be screened for relevance based on alignment with the study objectives and inclusion criteria. Only documents published by official government institutions and recognized international health organizations will be prioritized. A sample of search search strings will include Vaccin* OR immuniz*) AND Kenya; (routine vaccin* OR routine immuniz*) AND Kenya; (Vaccin* OR immuniz*) AND (vulnerab* OR marginalized) AND Kenya; (routine vaccin* OR routine immuniz*) AND ("Expanded Programme on Immunization" OR EPI OR KEPI).

 9. No mention of whether inter-rater reliability or agreement measures (e.g., Cohen’s Kappa) will be used. Author should clarify the criteria for resolving disagreements during screening as well as specify who will perform quality control and ensure consistency.

 Response: Whereas we don’t intend to use formal inter-rater reliability measures (e.g., Cohen’s Kappa in this review, the team will hold meetings to discuss the relevance of documents to be included for review and any disagreements addressed during the discussions.  

 10. The explanation of what constitutes “explicit and implicit” vulnerability-related information is vague. Author should define indicators of implicit vulnerability (e.g., mention of remote populations, nomadic groups, maternal illiteracy) and consider aligning categories with a policy analysis framework such as Walt & Gilson’s policy triangle.

 Response: We have spelt out the specific information on vulnerability that we intend to extract. This will be how vulnerability is defined, who is considered vulnerable and strategies to address the needs of those considered vulnerable. As for the use of a policy analysis framework such as Walt & Gilson’s policy triangle, in this review we only plan to synthesise the content of policy documents.

 11. The author should mention potential uses of findings (e.g., stakeholder engagement, policy briefs).

 Response: We have added information on how we intend to use the findings of the review. Our plan is to share the review findings with stakeholders through policy briefs and dissemination meetings and explore further through primary research focusing on vulnerabilities influencing childhood vaccination in Kenya.

 12. No mention of NVivo or qualitative coding software to manage complex themes. Author should consider using software like NVivo or Atlas.ti for theme development and outline how themes will be validated or revised.

 Response: We have included use of Nvivo software and the themes will be reviewed and revised through comparison across documents, and any discrepancies will be discussed amongst reviewers until a consensus is reached.  

 13. Author should add a paragraph in the discussion acknowledging this limitation e.g., exclusion of unpublished or draft policies.

 Response: We have outlined the biases we foresee in our review. These include: possibility of missing out on some of the documents that are not publicly available online and those still in the drafting stages. We also recognize that our findings will be limited to documents that fall within the inclusion criteria.

 14. **Conclusion: **Emphasizes intended use of findings to influence policy. Too generic. Does not outline how findings will be shared, with whom, and for what purpose. **Hence, **Author should add specifics: “We will share findings with the Kenya EPI taskforce, civil society actors, and Ministry of Health policymakers through workshops and briefings.”

 Response: In the revised conclusion, we have specified that we will share the findings through policy briefs and dissemination meetings with health and EPI stakeholders at national, county and sub-county levels.

**Areas needing revision**

 15. Conceptual Clarity: Define “vulnerability” more rigorously; use a guiding policy framework (e.g., WHO’s equity lens).

 Response: We have provided the following definition of vulnerability in the introduction section

 16. Search Strategy: Include a sample string and Google search filtering criteria.

 Response: This has been addressed by including a sample search string. We have also indicated that Google searches will be screened for suitability based on their alignment with review objectives and inclusion criteria. We will also only consider documents published by official government institutions and recognized international health organizations.

 17. Data Extraction & Coding: Add criteria for identifying implicit mentions; consider coding software.

 Response: We have specified the information we intend to extract from the documents and also added the use of NVivo software.

 18: Risk of Bias: Discuss selection bias from document inclusion and relevance thresholds.

 Response: Under the risk and bias section, we have indicated that potential risks of bias including missing unpublished or documents that are still being drafted and those that fall within the inclusion criteria (only English documents, and those published from 2000-2023).

 19. Conclusion: Specify dissemination pathways and intended policy impact more explicitly.

 We plan to share the findings through policy briefs and dissemination meetings with health and EPI stakeholders at national, county and sub-county levels to contribute to the improvement of the immunization policy landscape in Kenya.

# Reviewer report: Reviewer response for version 2

**DOI:** 10.3310/nihropenres.15217.r35834

**Published:** 2025-05-30

**Authors:** Patricia Mechael

**Affiliations:** 1 HealthEnabled, Cape Town, South Africa

This revision is much improved. The authors have sufficiently addressed the reviewer comments provided. I look forward to reading the study findings.

Is the study design appropriate for the research question?

Yes

Is the rationale for, and objectives of, the study clearly described?

Yes

Are sufficient details of the methods provided to allow replication by others?

Partly

Are the datasets clearly presented in a useable and accessible format?

Partly

Reviewer Expertise:

Immunization, digital health, data use, health equity

I confirm that I have read this submission and believe that I have an appropriate level of expertise to confirm that it is of an acceptable scientific standard.

# Reviewer report: Reviewer response for version 1

**DOI:** 10.3310/nihropenres.14588.r31141

**Published:** 2024-05-11

**Authors:** Nicholas Spencer

**Affiliations:** 1 University of Warwick, Coventry, England, UK

As the authors state, equity in vaccination uptake and coverage is essential in achieving the targets of the WHO agenda 2021-2030. The proposed review aims to explore the extent to which issues of (in)equity are included in Kenyan and Ugandan policy documents and identify gaps and opportunities presumably with a view to informing strategies to promote equity. I assume that the authors are aware of existing inequity in the two countries and relevant existing data should be included in the introduction to give the reader an indication of the extent of the problem. 

 Methodology used in conducting a systematic reviews of policy documents may differ in some respects from that required for a systematic review of published literature but clear inclusion/exclusion criteria and an appropriate measure of quality of the document would seem to me to be essential components of a robust review. The policy analysis triangle may provide a measure of quality but the authors need to specify how they will assess quality.

 This is a potentially valuable contribution to addressing vaccination inequity in these two countries but I think attention should be given to the issues I have raised.

Is the study design appropriate for the research question?

Partly

Is the rationale for, and objectives of, the study clearly described?

Yes

Are sufficient details of the methods provided to allow replication by others?

Partly

Are the datasets clearly presented in a useable and accessible format?

Not applicable

Reviewer Expertise:

Inequity in child health; Child Public Health including vaccination coverage issues

I confirm that I have read this submission and believe that I have an appropriate level of expertise to confirm that it is of an acceptable scientific standard, however I have significant reservations, as outlined above.

# Response

**Published:** 2025-05-19

**Authors:** Esther Owino

**Affiliations:** KEMRI Wellcome Trust Research Programme, Kenya

Thank you very much for the useful feedback.

 We have added the Inclusion criteria in the revised protocol.

 About the quality of the documents, we only aimed to examine the content of the documents in regard to vulnerability since the documents were not vulnerability-specific.

# Reviewer report: Reviewer response for version 1

**DOI:** 10.3310/nihropenres.14588.r31362

**Published:** 2024-05-11

**Authors:** Patricia Mechael

**Affiliations:** 1 HealthEnabled, Cape Town, South Africa

This proposed review of equity policies related to immunization is an important and timely piece of work. One recommendation is to not call it a "systematic review" as that has connotations of a state of evidence review based on randomized controlled trials. Rather it could be referred to as a "systematic policy review" or "systematic review of policies."

 A few additions that would strengthen the protocol would be the inclusion of a search strategy and/or initial coding structure, a proposed output of a timeline mapping number and type of equity-focused policies, and a proposed output of summarizing an equity-related immunization outcome (such as number/percent of zero-dose children) as available in existing surveys such as Demographic and Health Surveys and/or MICS. 

Is the study design appropriate for the research question?

Yes

Is the rationale for, and objectives of, the study clearly described?

Yes

Are sufficient details of the methods provided to allow replication by others?

Partly

Are the datasets clearly presented in a useable and accessible format?

Partly

Reviewer Expertise:

Immunization, digital health, data use, health equity

I confirm that I have read this submission and believe that I have an appropriate level of expertise to confirm that it is of an acceptable scientific standard, however I have significant reservations, as outlined above.

# Response

**Published:** 2025-05-19

**Authors:** Esther Owino

**Affiliations:** KEMRI Wellcome Trust Research Programme, Kenya

Thank you very much for this useful feedback.

 We have incorporated the recommendation about the title. The revised title now reads as follows; *Vulnerability and childhood immunization in Kenya: a systematic review of policy documents protocol*

 We have included the Inclusion and search strategy in the revised version.

 We have also included a data extraction template as one of our extended data files.

 Since we have revised the scope of the protocol and the focus is no longer on equity but vulnerability, the recommendation to map equity-based policies might not be applicable.
